# Rapid and Sensitive Detection of *Salmonella* spp. Using CRISPR-Cas13a Combined With Recombinase Polymerase Amplification

**DOI:** 10.3389/fmicb.2021.732426

**Published:** 2021-10-18

**Authors:** Bailin An, Hongbin Zhang, Xuan Su, Yue Guo, Tao Wu, Yiyue Ge, Fengcai Zhu, Lunbiao Cui

**Affiliations:** ^1^National Health Commission (NHC) Key Laboratory of Enteric Pathogenic Microbiology, Jiangsu Provincial Center for Disease Control and Prevention, Nanjing, China; ^2^College of Pharmacy, Nankai University, Tianjin, China; ^3^Jiangyin City Center for Disease Control and Prevention, Wuxi, China; ^4^School of Public Health, Nanjing Medical University, Nanjing, China

**Keywords:** *Salmonella*, foodborne disease, RPA, CRISPR-Cas13a, molecular detection

## Abstract

*Salmonella* spp. is one of the most common foodborne disease-causing pathogens that can cause severe diseases in very low infectious doses. Rapid and sensitive detecting *Salmonella* spp. is advantageous to the control of its spread. In this study, a conserved short fragment of the *Salmonella invA* gene was selected and used to design primers and specific crRNA (CRISPR RNA) for establishing a one-tube and two-step reaction system for *Salmonella* spp. detection, by combining recombinase polymerase amplification (RPA) with CRISPR-Cas13a (Clustered Regularly Interspaced Short Palindromic Repeats associated protein 13a) cleavage. The established one-tube RPA-Cas13a method can complete the detection within 20 min and the two-step RPA-Cas13a method detection time within 45 min. The designed primers were highly specific to *Salmonella* spp. and had no cross-reaction with the other nine diarrheal bacteria. The one-tube RPA-Cas13a could detect the *Salmonella* genome with the limit of 10^2^ copies, which was the same as real-time polymerase chain reaction (PCR), but less sensitive than two-step RPA-Cas13a (10^0^ copies). The detection results of one-tube or two-step RPA-Cas13a and real-time PCR were highly consistent in clinical samples. One-tube RPA-Cas13a developed in this study provides a simple, rapid, and specific detection method for *Salmonella* spp. While two-step assay was more sensitive and suitable for samples at low abundance.

## Introduction

*Salmonella*, a gram-negative bacillus, is the most common diarrheal pathogenic bacteria and infects millions of people across the world every year, with the most common clinical manifestations being acute gastroenteritis, encephalitis, pericarditis, sepsis, and even death (Hugas and Beloeil, 2014; Ferrari et al., 2019). According to statistics by the World Health Organization, the number of outbreaks of different food-borne intestinal diseases since 2010 has reached 582 million, with nearly 350,000 deaths, including 52,000 *Salmonella* cases (World Health Organization [WHO], 2016)]. In the United States, the Centers for Disease Control and Prevention (CDC) estimated food was one of the main sources of *Salmonella* infection; *Salmonella* caused approximately 1.35 million infections, 26,500 hospitalizations, and 420 deaths every year (CDC, 2020). In the European Union, European Centre for Disease Prevention and Control reported 91,857 *Salmonella* infection cases in humans, and *Salmonella* caused 30.7% of all food-borne outbreaks during 2018 (European Food Safety Authority, & European Centre for Disease Prevention and Control, 2020). In China, the previous study has estimated the incidence of non-typhoid salmonellosis at 626.6 cases per 100,000 people (Yue et al., 2020). Timely screening of *Salmonella* is the key to prevent and control diarrheal disease outbreaks. To date, the detection methods for *Salmonella*, including traditional biochemical culture, immune testing, and molecular biological approaches, are represented by polymerase chain reaction (PCR)/real-time PCR. These methods are time-consuming, or poor in specificity or of low sensitivity, and sometimes require expensive instruments for laboratory setup. Especially, many diarrheal pathogenic bacteria, including *Salmonella*, can cause severe infectious diseases at low infectious doses. More sensitive and specific *Salmonella* detection methods are urgently needed.

Over the past year, researchers have applied the latest recombinase polymerase amplification (RPA)/recombinase aided amplification (RAA) techniques to achieve amplification for *Salmonella*. However, the detection of amplification products by agarose gel electrophoresis showed poor sensitivity, and fluorescent probes for visual detection were expensive. A typical detection of pathogenic *Salmonella* was reported by Zhang et al. (2017) using the real-time fluorescence RAA detection system, but the detection limit was 10^3^ copies/μL, equivalent to ∼5 fg plasmid DNA. An optical biosensor reported by Zheng et al. (2020) could detect 1.8 × 10^1^ copies/μL but was not useful in POCT (point-of-care testing).

The CRISPR-Cas (Clustered Regularly Interspaced Short Palindromic Repeats-CRISPR associated protein) system is a sophisticated adaptive immune system, and the different CRISPR-Cas nucleases display specific cleavage activity when target-specific crRNA recognizes the DNA target (Bhaya et al., 2011; Jackson et al., 2014; Kirchner and Schneider, 2015). In recent years, researchers have found that the cleavage activity of CRISPR-Cas nucleases could be used not only as a programmable tool for gene editing but also for *in vitro* nucleic acid detection (Arslan et al., 2014; Zhou et al., 2020). In this study, we established a one-tube and a two-step reaction system for *Salmonella* spp. detection by combining RPA with CRISPR-Cas13a cleavage, which provided a sensitive and convenient molecular detection method for the early diagnosis of diarrheal diseases of *Salmonella* infection.

## Materials and Methods

### Cas13a Protein Expression and Purification

Cas13a was purified as referred to in previous studies (East-Seletsky et al., 2016, 2017; Su et al., 2020). Briefly, the Cas13a gene in plasmid pC019-LwCas13a from *Leptotrichia wadei* (Addgene, United States) was subcloned into the psmarti vector (*xhoI* restriction site), and the correct plasmid was transformed into BL21(DE3) (General Biosystem, China). The expression of the target protein was induced at different temperatures (15 and 37°C) and different concentrations of IPTG (0.2 and 1.0 mM) (Amresco, United States). The Cas13a protein was purified by Ni-NTA (Smart-Lifesciences, China) using the 6 × His Tag antibody and horseradish peroxidase conjugate (Invitrogen, United States). After the addition of SUMO Protease (General Biosystem, China) to remove the fusion SUMO label, and the purified target protein was dialyzed into protein buffer [50 mM Tris, pH 7.5, 600 mM NaCl, 5% (vol/vol) glycerol, and 2 mM DTT].

### Strains, Clinical Samples, and Nucleic Acid Extraction

*Salmonella* and other diarrheal pathogenic bacteria (*Staphylococcus aureus*, *Listeria monocytogenes*, *Enterococcus faecalis*, *Shigella flexneri*, *Vibrio parahemolyticus*, *Escherichia coli* O157, *Yersinia enterocolitica*, *Campylobacter jejuni*, *Vibrio vulnificus*) were provided by Jiangsu Provincial Center for Disease Control and Prevention. A total of 84 clinical stool samples that were selected for clinical validation of the method were collected from different hospitals in Jiangsu Province in 2019. The nucleic acid was extracted by EX-DNA Bacterial Genomic Extraction Kit (Tianlong, China) on a fully automatic nucleic acid extractor (Tianlong, China).

### Preparation of Primers, Probes, and crRNA

*Salmonella*-specific *invA* gene was retrieved from GenBank, and the conservative sequence region was selected to design RPA amplification primers using Primer 5 software, and Primer-BLAST software was used to verify the specificity of the primer sequence. The designed primers, probes, and oligonucleotides were synthesized by Sangon Biotech (Shanghai, China). The oligonucleotides containing T7 promoter, repeat, and spacer sequences were annealed with a T7 primer. Then, the crRNA was synthesized by incubating at 42°C for 2 h with T7 RNA polymerase (TaKaRa, China). The synthesized crRNA was digested with DNase I (TaKaRa, China) at 37°C for 1 h followed by purification with RNA rapid concentration and purification kit (Sangon Biotech, China) according to the manufacturer’s protocol. The concentration of crRNA was quantified using Qubit 2.0 (Invitrogen, United States). All nucleic acid sequences used in this study are shown in Table 1.

**TABLE 1:** RPA primers, crRNA, and RNA reporter probe sequences.

**Name**	**Sequences(5′–3′)**
*Salmonella* RPA-F	TGTTGTCTTCTCTATTGTCACCGTGGTCCAG
*Salmonella* RPA-R	TAATACGACTCACTATAGGGTAC CGGGCATACCATCCAGAGAAAATCGGGCCGC
*Salmonella* crRNA	GACACGTTCTGAACCTTTGGTAA TAACGGTTTTAGTCCCCTTCGTTTTTGGGGTA GTCTAAATCCCTAT AGTGAGTCGTATTA
RNA probe	FAM-TrUrUrUrUrUrC-BHQ1

### Collateral Cutting Capacity of CRISPR-Cas13a

The amplicon of the RPA reaction was used as the template for transcription *in vitro* with T7 RNA polymerase, and the transcription product was purified to obtain the *Salmonella* target RNA. Then, the target RNAs were diluted gradiently (10^0^–10^5^ pM). The synthesized oligonucleotide *Salmonella*-RPA-R was used as a non-target control (500 ng/μL). Verification of CRISPR-Cas13a cutting activity was completed as follows: 5 × reaction buffer 5 μL (final concentration 20 mM HEPES, 60 mM NaCl, 6 mM MgCl_2_, pH 6.8), 25 mM NTP mix 1 μL (ATP, GTP, CTP, UTP, Bio Lab, China), Recombinant RNase Inhibitor (TaKaRa, China) 0.5 μL, 1.2 μM crRNA 0.6 μL, 10 μM RNA-probe 0.5 μL, 1 μM Cas13a 1 μL, or 10^3^ pM target RNA or non-target control 2 μL, adding RNase-free ddH_2_O to 25 μL. The reaction was conducted at 39°C for 30 min, and RNase A was used as the positive control.

### Two-Step RPA-Cas13a

In the two-step RPA-Cas13a assay, basic RPA reactions were first conducted according to the instructions of the TwistAmp Basic Kit (Twist Dx, Cambridge, United Kingdom). Each RPA reaction was carried out in a 50 μL reaction volume containing Primer Free Rehydration buffer 29.5 μL, 0.24–0.96 μM forward and reverse primers each 0.5 μL, target DNA template 2 μL, and RNase-free ddH_2_O 15 μL. The reaction mixes were vortexed and spun briefly, and then 280 mM magnesium acetate (MgOAc) 2.5 μL was added and mixed well to start a reaction in fluorescence detector F1620 (Qitian, China) at 37, 39, and 41°C for 20 min. Then, a 25 μL CRISPR-Cas13a reaction system was performed with 5 × reaction buffer 5 μL, 25 mM NTP mix 1 μL, Recombinant RNase Inhibitor 0.5 μL, 1.2 μM *Salmonella*-crRNA 0.6 μL, 10 μM RNA-probe 0.5 μL, T7 RNA Polymerase 0.3 μL, 1 μM Cas13a 1 μL, and added RNase-free ddH_2_O to 23 μL, and RPA products 2 μL. The fluorescent signals were collected at 39°C for 30 min in fluorescence detector F1620.

### One-Tube RPA-Cas13a

The one-tube RPA-Cas13a assay combined RPA with CRISPR-Cas13a in a one-tube reaction system. Briefly, the 50 μL one-tube reaction system consisted of Primer Free Rehydration buffer 29.5 μL, 25 mM NTP mix 4 μL, Recombinant RNase Inhibitor 4 μL, T7 RNA Polymerase 1 μL, 0.24–0.96 μM primers each 0.5 μL, 1.2 μM crRNA 1 μL, 10 μM RNA-probe 1 μL, 1 μM Cas13a 2 μL, target DNA template 2 μL, and RNase-free ddH_2_O 1 μL. The reaction mixes were vortexed and spun briefly, and then 280 mM MgOAc 2.5 μL was added to the tube cap and was centrifuged into the reaction solution. Fluorescent signals were collected at 37, 39, and 41°C for 2 h in fluorescence detector F1620.

### Sensitivity and Specificity of One-Tube and Two-Step RPA-Cas13a

To evaluate the minimum detection limit, serial dsDNA standards were prepared as follows: using the extracted nucleic acid of *Salmonella* as a template, a 50 μL LA Taq PCR (TaKaRa, China) containing LA Taq 0.5 μL, 2.5 μM dNTP 4 μL, 10 × buffer 5 μL, 10 μM forward/reverse primer each 1 μL, and RNase-free ddH_2_O 37.5 μL was performed. The PCR amplification product was purified by High Pure PCR Product Purification Kit (Roche, CH). The dsDNA quantification was performed in a Qubit digital fluorimeter using the Qubit dsDNA BR Assay (Invitrogen, Waltham, United States). The dsDNA copy number was then determined using the following formula: {[6.02 × 10^14^ × dsDNA concentration (ng/μL) × 10^–9^]}/[DNA in length × 660]. The gradiently diluted dsDNA standards (10^7^∼10^–1^ copies/μL) were detected by the one-tube and two-step RPA-Cas13a. We also chose real-time PCR (Hein et al., 2006) (TaKaRa, China) as the reference test. The specificity of the one-tube and two-step RPA-Cas13a was tested by using nucleic acid extracted from diarrheal pathogenic bacteria strains: *Salmonella*, *S. aureus*, *L. monocytogenes*, *E. faecalis*, *S. flexneri*, *V. parahemolyticus*, *E. coli* O157, *Y. enterocolitica*, *C. jejuni*, and *V. vulnificus*.

### Real-Time Polymerase Chain Reaction

Real-time PCR was performed on the LightCycler 2.0 instrument in which premix Ex Taq^TM^ (Takara, China) was used (Hein et al., 2006). The reaction was performed as follows: 95°C for 30 s, followed by 40 cycles of 95°C for 5 s and then 60°C for 20 s.

### Consistency Comparison Using Real-Time Polymerase Chain Reaction

Genomic DNAs from 84 clinical stool samples were selected for a clinical comparison experiment. All samples were tested with the two-step, one-tube RPA-Cas13a, and real-time PCR assay (Hein et al., 2006). The consistency of the three methods above was compared using SPSS 24.0 software. *κ*-tests were used for consistency analysis.

## Results

### Collateral Cutting Capacity of CRISPR-Cas13a

**FIGURE 1:**
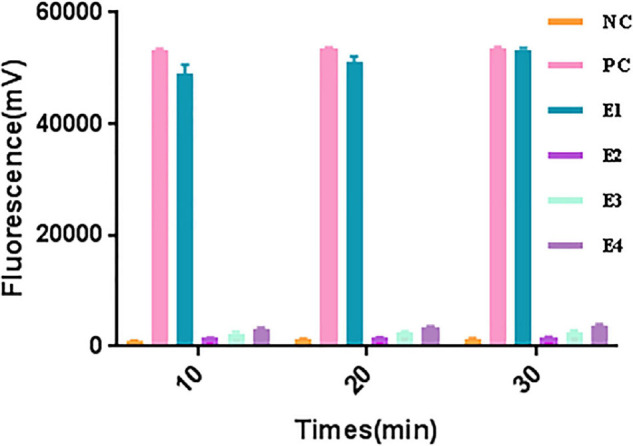
The collateral cutting activity of CRISPR-Cas13a. NC, negative control (RNase-free ddH_2_O); PC, positive control (RNase A); E1, Cas13a with 10^3^ pM target RNA; E2, Cas13a with 10^3^ pM non-target control; E3, Cas12a with 10^3^ pM target RNA; E4, Cas13a with non-crRNA.

**FIGURE 2:**
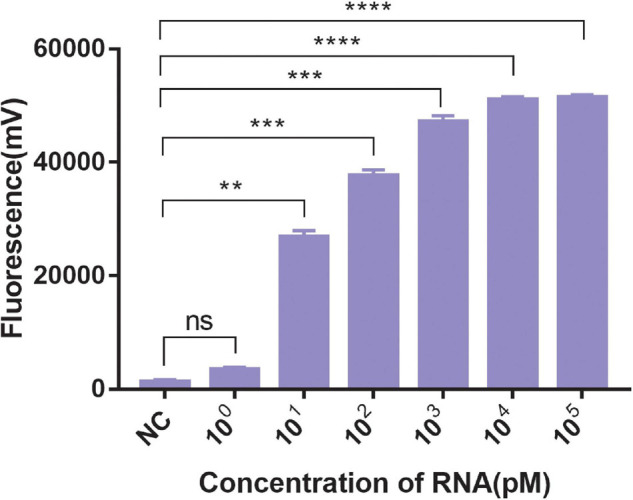
The sensitivity of CRISPR-Cas13a. 10^0^∼10^5^ pM were the concentrations of RNA standards. NC, negative control (RNase-free ddH_2_O). Unpaired two-tailed *t*-test was used to analyze the difference from NC. **p < 0.01; ***p < 0.001; ****p < 0.0001. ns, not significant.

A significant fluorescent signal in the reactions of the positive control within was verified with RNase A. Compared with the detection of Cas13a for target RNA and non-target RNA control, the results showed that Cas13a could achieve specific detection for target RNA of *Salmonella* in the presence of its corresponding crRNA. Furthermore, there was no detection signal generated for Cas12a compared to Cas13a. The detection signal of CRISPR-Cas13a for 10^3^ pM was almost identical to that of the positive control (Figure 1). The results of different dilutions for RNA standards showed that the minimum test limit was 10 pM (Figure 2).

### Optimization of Two-Step RPA-Cas13a Detection System

**FIGURE 3:**
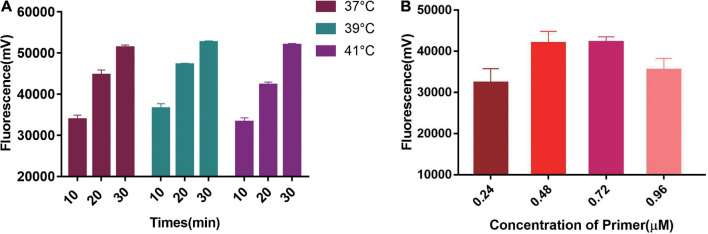
Single factor screening results of two-step RPA-Cas13a detection. **(A)** The effect of temperatures, 37, 39, and 41°C represented the temperature of the RPA reaction. **(B)** The effect of RPA primer concentration.

We first tested the best RPA amplification temperature using 0.48 μM primer concentration. The results showed the highest fluorescent signals were obtained at 39°C in all three Cas13a detecting time points (Figure 3A). Therefore, 39°C was selected as the best RPA amplification temperature. Then, the best primer concentrations were explored at 39°C. The optimal primer concentration of 0.48 μM was found (Figure 3B). Therefore, we chose 0.48 μM and 39°C as the best RPA primer concentration and amplification temperature.

### Optimization of One-Tube RPA-Cas13a Detection System

**FIGURE 4:**
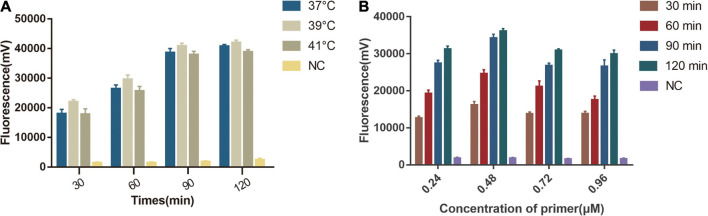
Single factor screening results of one-tube RPA-Cas13a detection. **(A)** The effect of temperature on one-tube RPA-Cas13a. 37, 39, and 41°C represented the temperature of the one-tube RPA-Cas13a reaction. NC, negative control (RNase-free ddH_2_O). **(B)** The effect of primer concentration on one-tube RPA-Cas13a. 30, 60, 90, and 120 min represented the time of one-tube RPA-Cas13a reaction. NC, negative control (RNase-free ddH_2_O).

We first tested the different temperature (37, 39, 41°C) in a one-tube RPA-Cas13a detection system and found that 39°C had the highest fluorescent signals. The detection signal grew stronger from 10 to 90 min and reached its peak at 90 min, whereas the negative control had no visible detection signal (Figure 4A). We then explored the optimal primer concentration under 39°C and found the highest fluorescent signals were obtained at 0.48 μM primer concentrations. Therefore, the optimized conditions of one-tube RPA-Cas13a were 39°C with 0.48 μM primer concentration (Figure 4B).

### Sensitivity and Specificity of One-Tube and Two-Step RPA-Cas13a

**FIGURE 5:**
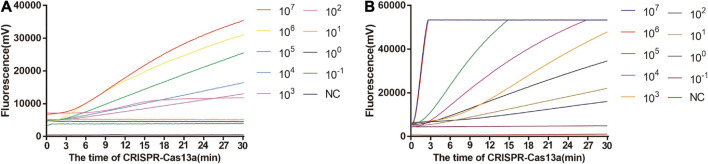
The sensitivity of one-tube and two-step RPA-Cas13a. **(A)** The sensitivity of one-tube RPA-Cas13a. **(B)** The sensitivity of two-step RPA-Cas13a. 10^7^∼10^– 1^ (copies/μL) represented the concentration of *Salmonella* dsDNA standards. NC, negative control (RNase-free ddH_2_O).

**FIGURE 6:**
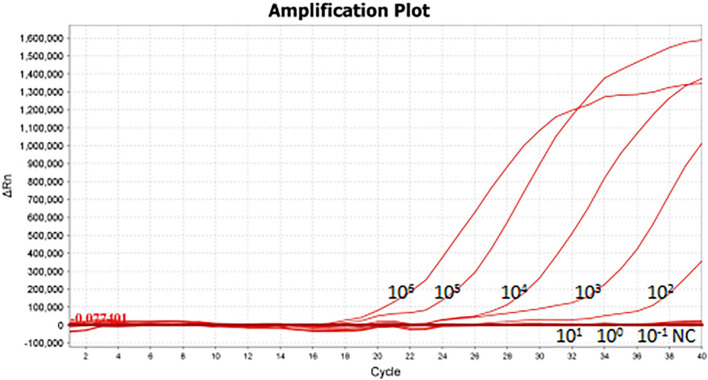
The sensitivity of real-time PCR 10^6^∼10^– 1^ (copies/μL) represented the concentration of *Salmonella* dsDNA standards. NC, negative control (RNase-free ddH_2_O).

**FIGURE 7:**
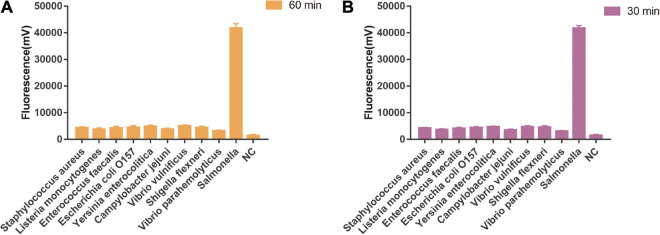
The specificity of one-tube and two-step RPA-Cas13a. **(A)** The specificity of one-tube RPA-Cas13a. 60 min was the time of one-tube RPA-Cas13a. NC, negative control (RNase-free ddH_2_O). **(B)** The specificity of two-step RPA-Cas13a. 30 min was the time of CRISPR-Cas13a reaction. NC, negative control (RNase-free ddH_2_O).

As shown in Figure 5A, the limit of detection of one-tube RPA-Cas13a for *Salmonella* was 10^2^ copies/μL, which showed the same sensitivity to real-time PCR (10^2^ copies/μL, Figure 6) and slightly lower than that of two-step RPA-Cas13a (10^0^ copies/μL, Figure 5B). The whole detection time of one-tube RPA-Cas13a for 10^2^ copies/μL was only 20 min (Figure 5A), which was shorter than those for two-step RPA-Cas13a (RPA: 20 min, transfer time: 5 min, and CRISPR-Cas13a: 20 min, Figure 5B) and real-time PCR (60 min or much longer, Figure 6). For specificity assessment, whether one-tube RPA-Cas13a at 60 min (Figure 7A) or two-step RPA-Cas13a at 30 min (Figure 7B), only the *Salmonella* showed detection signals among the 10 common diarrheal bacteria, and no cross-reactions were found.

### Consistency Comparison Using Real-Time Polymerase Chain Reaction

For evaluating the applicability of developed one-tube and two-step RPA-Cas13a for *Salmonella* in clinical stool samples, a total of 84 clinical stool samples from different hospitals were selected and tested by real-time PCR, two-step RPA-Cas13a, and one-tube RPA-Cas13a. Sixteen samples were detected positive for *Salmonella* by real-time PCR. Besides those 16 samples, three additional samples were judged as positive by two-step RPA-Cas13a in which one was also detected positive by one-tube RPA-Cas13a. However, the signal intensity was significantly lower than that of the other 16 positive samples but significantly higher than that of the negative control sample. At the same time, those three samples were also identified by Sanger sequencing technology and determined to be positive for *Salmonella*. The two-step RPA-Cas13a detection results are in good consistency with real-time PCR (*κ* = 0.892), the one-tube RPA-Cas13a detection results are also in good consistency with real-time PCR (*κ* = 0.962), and the statistical results are shown in Table 2.

**TABLE 2:** Clinical samples consistency comparison using three detection methods.

		**Real-time PCR result**		**Total**	**Sensitivity (%) (95% CI)**	**Specificity (%) (95% CI)**	***κ***
		**+**	**−**				
One-tube RPA-Cas13a	+	16	1	17	100	98.50	0.962
	−	0	67	67			
	Total	16	68	84			
Two-step RPA-Cas13a	+	16	3	19	100	95.60	0.892
	−	0	65	65			
	Total	16	68	84			

*+, Positive; −, negative; CI, confidence interval. Data determined by SPSS 24.0 software. One-tube RPA-Cas13a–positive result, 17; one-tube RPA-Cas13a–negative result, 67; two-tube RPA-Cas13a–positive result, 19; two-tube RPA-Cas13a–negative result, 65; real-time PCR-positive result, 16; real-time PCR-negative result, 68.*

## Discussion

Currently, the isothermal amplification of nucleic acid technology has been used for the rapid pathogen detection of infectious diseases because of its high specificity, efficiency, and simplicity and plays an increasingly important role in promoting field detection and control of pathogen diseases. But the isothermal amplification of nucleic acid technology has its disadvantages, such as the LAMP (loop-mediated isothermal amplification) having an intricate primer design and non-specific reactions. Compared with other isothermal amplification technologies, RPA/RAA can be performed near ambient temperature (37–42°C) and has higher amplification efficiencies in recent years. The RPA process relies on three core enzymes, the recombinant enzymes uvsX and uvsY, SSB gp32 (Single-stranded DNA-binding protein), and Bsu DNA polymerase helping target regions of the template that can exponentially amplify within 20 min (Ahmed et al., 2015; Ma et al., 2019). Most importantly, the RPA/RAA has a fairly simple primer design. Commonly, the detection platforms most used for post-RPA detection are fluorescence RPA/RAA, gel electrophoresis, or LFD (lateral flow dipstick), but these methods require an expensive design for the probe, low sensitivity, or lack of specificity. It is necessary to establish a more sensitive and specific detection method that can be combined with RPA.

In recent years, studies have found that the collateral cutting activity of CRISPR-Cas13a is activated by a specific crRNA that targets a specific single-stranded RNA sequence, and collateral cutting activity can then cleave nearby non-specific RNAs (Knott et al., 2017; Zuo et al., 2017). Based on this principle, non-specific RNA reporting probes can add to the reaction system to achieve specific detection of target molecules (Abudayyeh et al., 2016; Gootenberg et al., 2017). The feature of collateral cutting activity has been used in the field of nucleic acid detection (Shen et al., 2020), among which the most representative is the SHERLOCK (Specific High-sensitivity Enzymatic Reporter UnLOCKing) by combining RPA with CRISPR-Cas13a (Myhrvold et al., 2018). This method provides a new direction for developing nucleic acid detection technology for pathogenic microbes, with the advantages of simplicity, rapidity, sensitivity, and specificity. The current detection based on SHERLOCK is mainly a two-step method. The one-tube method has been reported, but its sensitivity is insufficient.

The T7 promoter sequence is introduced into the RPA amplification primer, and the RPA amplification products are transcribed into a large amount of RNA by T7 RNA polymerase, which improves the sensitivity of detecting *Salmonella* targets. The results showed that two-step RPA-Cas13a is more sensitive than real-time PCR. Its detection limit for *Salmonella* can reach 10^0^ copies/μL, One-tube RPA-Cas13a could achieve the same detection sensitivity as real-time PCR with 10^2^ copies/μL. In addition, the crRNA design of CRISPR-Cas13a is relatively simple, requiring only 24 bases. The target-specific crRNA would further increase the detection specificity. The specificity results showed that there is no cross-reaction between the RPA-Cas13a of *Salmonella* and the other nine diarrheal pathogenic bacteria. Compared with the fluorescence probes used in RPA/RAA assay, the CRISPR non-specific detection probe is pretty easy to design in the present study and cost saving. In addition, both RPA and CRISPR detection can be conducted on a simple constant temperature equipment, which greatly reduces the requirements for equipment and personnel. The one-tube RPA-Cas13a detection process can detect *Salmonella* within 20 min, while two-step RPA-Cas13a costs 45 min, which was shorter compared with LAMP and real-time PCR method. The influence of clinical samples on RPA-Cas13a detection performance was verified: the *Salmonella* detection rate of the two-step or one-tube RPA-Cas13a detection method was highly consistent as that of real-time PCR and could even detect clinical stool samples at low infectious doses, whereas three samples with low doses cannot be detected by real-time PCR. Of the three low infectious dose samples, one can detect by one-step assay, and three can detect by two-step assay. Although the two-step RPA-Cas13a assay is more sensitive, it is also more easily contaminated. Some intervention measures, such as using oily non-interfering substances to seal the liquid surface, the use of specific reagent tubes, and so on, can be used to reduce or avoid the contamination, which would help this assay to be used in routine clinical trials. We recommend one-tube RPA-Cas13a assay as the rapid POCT method for *Salmonella* in rural hospitals with resource-poor settings, whereas two-step RPA-Cas13a for low abundance detection.

## Conclusion

In this study, a novel molecular diagnostic method based on CRISPR-Cas13a combined with RPA was successfully demonstrated for rapid, sensitive, and specific detection of *Salmonella* and provides a new approach for the rapid screening of clinical samples in hospitals.

## Data Availability Statement

The raw data supporting the conclusions of this article will be made available by the authors, without undue reservation.

## Ethics Statement

The studies involving human participants were reviewed and approved by the Jiangsu Provincial Center for Disease Control and Prevention. The ethics committee waived the requirement of written informed consent for participation.

## Author Contributions

BA: methodology establishment, data sorting, and analysis, visualization, writing—original draft. HZ: methodology establishment, data sorting, writing—review and editing, and software. XS: methodology establishment, data sorting, and analysis. YuG and TW: methodology establishment. YiG: funding acquisition and writing—review and editing. FZ: project administration and supervision. LC: conceptualization, funding acquisition, project administration, validation, and writing—review and editing. All authors contributed to the article and approved the submitted version.

## Conflict of Interest

The authors declare that the research was conducted in the absence of any commercial or financial relationships that could be construed as a potential conflict of interest.

## Publisher’s Note

All claims expressed in this article are solely those of the authors and do not necessarily represent those of their affiliated organizations, or those of the publisher, the editors and the reviewers. Any product that may be evaluated in this article, or claim that may be made by its manufacturer, is not guaranteed or endorsed by the publisher.
